# Iron-Mediated Overexpression of Amyloid Precursor Protein via Iron Responsive mRNA in Alzheimer’s Disease

**DOI:** 10.3390/ijms26115283

**Published:** 2025-05-30

**Authors:** Mateen A. Khan

**Affiliations:** Department of Life Science, College of Science and General Studies, Alfaisal University, Riyadh 11533, Saudi Arabia; matkhan@alfaisal.edu

**Keywords:** Alzheimer’s disease, iron overload, iron-responsive element, iron regulatory protein, amyloid precursor protein, amyloid-β

## Abstract

Iron accumulation in the brain is widespread in Alzheimer’s disease (AD), the most common cause of dementia. According to numerous studies, too much iron triggers the development of neurofibrillary tangles (NFTs) and amyloid-β (Aβ) plaques, both of which accelerate the onset of AD. Iron sequestration and storage were disrupted by high iron, and the pattern of interaction between iron regulatory proteins (IRPs) and iron-responsive elements (IREs) was altered. The 5′-untranslated regions (5′-UTRs) of their APP mRNA transcripts have an IRE stem-loop, which is where iron influx enhances the translation of the amyloid precursor protein (APP). Iron regulated APP expression via the release of the repressor interaction of APP mRNA with IRP1 by a pathway similar to the iron control translation of the ferritin mRNA by the IREs in their 5′-UTRs. This leads to an uncontrolled buildup of redox active Fe^2+^, which exacerbates neurotoxic oxidative stress and neuronal death. Fe^2+^ overload upregulates the APP expression and increases the cleavage of APP and the accumulation of Aβ in the brain. The level of APP and Aβ, and protein aggregates, can be downregulated by IRPs, but are upregulated in the presence of iron overload. Therefore, the inhibition of the IRE-modulated expression of APP or Fe^2+^ chelation offers therapeutic significance to AD. In this article, I discuss the structural and functional features of IRE in the 5′-UTR of APP mRNA in relation to the cellular Fe^2+^ level, and the link between iron and AD through the amyloid translational mechanism. Although there are currently no treatments for AD, a progressive neurodegenerative disease, there are a number of promising RNA inhibitor and Fe^2+^ chelating agent therapeutic candidates that have been discovered and are being validated in April 2025 clinical trials. Future studies are expected to further show the therapeutic efficacy of iron-chelating medications, which target the APP 5′-UTR and have the ability to lower APP translation and, consequently, Aβ levels. As a result, these molecules have a great deal of promise for the development of small-molecule RNA inhibitors for the treatment of AD.

## 1. Introduction

Alzheimer’s disease (AD) is the most prevalent type of dementia and a neurological illness. Extracellular amyloid-β (Aβ) plaques and intracellular tau-enriched neurofibrillary tangles (NFTs) are two hallmarks of Alzheimer’s disease (AD). The essential protein implicated in the pathophysiology of AD, the amyloid precursor protein (APP), is the source of the Aβ-peptide [1,2]. Amyloidogenic or non-amyloidogenic pathways involving physiological and pathological processes are used to post-translate APP. Abundant Aβ-peptide and NFTs, together with elevated brain iron levels, are pathological hallmarks of AD in the brain cortex [3]. Overdosing on iron leads to the build-up of Aβ and the creation of NFTs, both of which accelerate the progression of AD [4]. These Aβ plaques and neuro-fibrillations eventually lead to neurodegeneration and neuronal death [5,6]. The misfolded phosphorylated tau protein in NFTs and misfolded Aβ-peptide accumulated in senile plaques cause memory loss and confusion, which eventually leads to personality and cognitive decline [7,8]. The primary constituent of these plaques is the Aβ_1–42_ peptide, which is cleaved from the APP, a trans-membrane metalloprotein [9].

Elderly people are more prone to develop AD, and aging is still thought to be the main risk factor for the condition [10,11]. Age-related increases in brain iron buildup vary by anatomical location [12,13], and AD patients have iron accumulation in insoluble amyloid plaques and NFTs [14]. On the other hand, one possible pathogenic reason for dementia is iron deposition in the brain. Various iron complexes build up in brain areas linked to cognitive and motor dysfunction as people age. Cellular iron accumulation and distribution are changed in AD due to alterations in iron homeostasis. Selective iron buildup takes place in many brain regions and cell types during healthy aging, with iron mostly bound within ferritin and neuromelanin [15,16,17]. Nevertheless, AD is linked to oxidative stress and cellular damage, and it is characterized by an accumulation of iron in certain brain regions that is higher than that observed in healthy aging. It is not evident if the iron buildup observed in AD is a main cause or a subsequent consequence. Using magnetic resonance imaging, it was discovered that AD patients’ brains had a markedly elevated iron level [18]. A further study that compared APP/PS1E9 double transgenic AD mice with wild-type mice of the same age supported this conclusion [19]. The globus pallidus, substantia nigra, and basal ganglia all contain high quantities of iron, and autopsy data indicate that total iron accumulation in the human brain is positively correlated with age [20].

Moreover, the effect of genetic hemochromatosis on iron accumulation in the central nervous system is still disputed. A recent study found a significant correlation between high brain iron levels and the hemochromatosis genotype [21]. However, iron deposition is localized to motor circuits of the brain; therefore, hemochromatosis may increase the risk of neurological movement disorders, such as Alzheimer’s. Notably, different brain areas are affected in these two pathological conditions [22]. Hereditary hemochromatosis is linked to a higher risk of dementia among male homozygotes who have the p.C282Y mutation in the homeostatic iron regulator (HFE) gene [23], but not a higher risk of AD specifically, as compared to non-carriers [24]. Additionally, it has been demonstrated that dementia liability is also linked to genetically determined serum iron (ferritin and transferrin saturation [TSAT]) in the general population. A causal relationship between iron and dementia in a community cohort is further supported by these investigations. A hallmark of cognitive decline is gray matter shrinkage, and research [25] suggests that plasma iron plays a part in this dementia-causing pathway. It is unknown if genetic variants linked to greater iron have a causal association with non-AD dementias in the general population, whereas genetic variants linked to plasma iron are not linked to the risk of AD in the general population [26]. The biology of metal (Fe) has been connected to brain aging and major neurodegenerative diseases, such as AD, by genetic and molecular evidence [27]. It has been reported that the disruption of the metal steady state in neurons can exacerbate neurodegenerative disease [28,29].

APP is a crucial membrane protein that is widespread in expression and mostly found in neuronal synapses. In addition to its role as a cell surface receptor, it has been linked to the regulation of iron export, synapse formation, brain plasticity, and antivirus activity. The many derivatives that are produced by different enzymatic processes are known to have different biological activities. By affecting neural stem cell proliferation, cell fate specification, and neurogenesis, APP may have a significant impact on neural growth and maturation during brain development. Two mutually exclusive pathways, the non-amyloidogenic pathway and the amyloidogenic pathway—are the primary means by which the proteolytic cleavage of APP takes place. Aβ-peptides, soluble APPα (sAPPα), soluble APPβ (sAPPβ), and the APP intracellular domain are among the various products produced by these pathways. Their functions are dictated by their structural variations, equilibrium, and concentration in relation to other APP fragments [30]. The most well-known function of APP is as the precursor molecule that is broken down by β-secretases and γ-secretases to produce the toxic Aβ peptide (Aβ_40_ and Aβ_42_) that damages brain neurons; this process is increased when iron is in excess [31].

The iron response element (IRE) in the 5′-untranslated region (5′-UTR) of the APP transcript regulates APP expression and Aβ production in response to intracellular iron levels [8]. Similar to how iron controls the translation of ferritin messenger RNAs (mRNAs) by the IREs in their 5′-UTRs, iron also tightly controls the expression of the Alzheimer’s APP gene at the level of message translation. Iron regulatory proteins (IRPs), which attach to the IREs found in the 5′-UTRs of respective mRNA transcripts, post-transcriptionally regulate ferritin and APP. The overexpression of amyloid and its aggregation on and in neurons are caused by the IRP/IRE signaling system, which is imbalanced when iron dysregulation occurs inside cells and affects IRP1’s attachment to the IRE [32]. A study reported that APP can bind to the iron exporter ferroportin, thus possibly facilitating iron export.

Metal ions such as iron (Fe), copper (Cu), and zinc (Zn) are essential for several brain activities, such as signal transmission, neurotransmitter synthesis, neuronal myelination, and defense against reactive oxygen species [33]. It is evident that these metals supply one of the ultrastructural prerequisites for Aβ peptide polymerization [34]. It was shown that metals played a crucial role in mediating the neurotoxic effects of Aβ protofibrils. According to the report, AD is associated with oxidative stress, neuronal oxidative damage, and brain metal imbalance. Elevated steady-state levels of iron in AD cause a pattern of gene expression linked to detrimental effects on neuronal survival. RNA oxidation and other types of oxidative damage, as well as the disruption of iron homeostasis, are supported by many studies on AD [35]. The metal hypothesis, put forth by Bush and Tanzi, states that Aβ–metal interactions may even be necessary for the neuropathogenic consequences of Aβ in AD [36]. The iron-mediated APP mRNA translational control and copper-mediated APP gene transcriptional control both offer fresh genetic evidence in favor of the theory that APP is a metalloprotein that plays a crucial part in metal metabolism [37,38].

The purpose of this review is to discuss recent findings on iron toxicity and possible connections between Aβ aggregation, APP translation and processing dysregulation, and iron dysregulation in the brain. For continuity and comprehension, I also direct the reader to a number of recent primary research and review papers (April 2025, search engine: https://pubmed.ncbi.nlm.nih.gov) for a thorough summary of earlier research findings. Select research studies by various groups of researchers within diverse disciplines. Conduct searching in stages by reading abstracts first and making selections and then reading full-text articles. Moreover, the collected text was screened in full to ensure the article meets the inclusion criteria. Furthermore, the references in the selected articles used to be scanned to identify other articles that were potentially relevant to the review. I start by giving a quick overview of the structure and function of IREs’ mRNA, current research on the binding mechanism of IREs/IRP, and the role of iron in complex formation. A brief explanation of how iron contributes to APP overexpression and the regulation of APP mRNA translation follows. Additionally, I provide an update on the processes governing brain iron homeostasis and a deeper comprehension of the current state of iron ions in amyloid overexpression in AD. Therapies including metal chelation and small compounds that either directly or indirectly disrupt Aβ-induced toxicity are also covered. It also offers the route for the build-up of neurotoxic Aβ of AD, which could help create new RNA-based medicinal compounds that impede the progression of APP or Aβ in AD.

## 2. Structure and Function of IREs

### 2.1. IRE Structure

**Figure 1:**
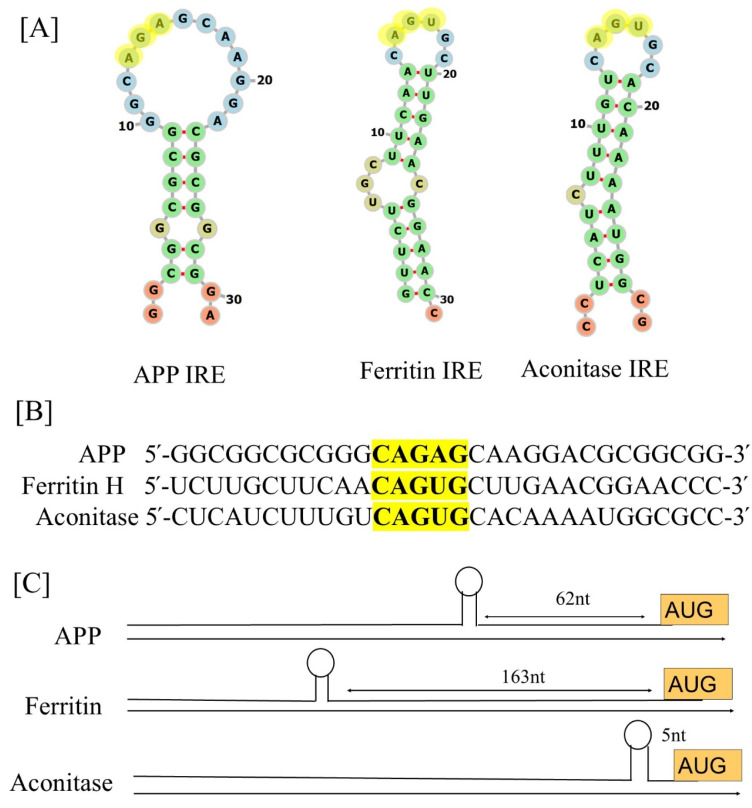
Comparison of the 5′-UTR IRE stem-loops in the mRNAs of the APP, ferritin-H, and mitochondrial aconitase transcripts. (**A**) Potential iron-regulating protein binding motifs and IRE-like AGU/AGA tri-loops (highlighted in yellow letters) have been identified in important transcripts associated with neurodegenerative diseases, including human APP, ferritin-H, and mitochondrial aconitase transcripts. (**B**) Sequences encoding the 5′-UTR-specific IRE stem-loops in an APP transcript aligned with those of ferritin-H and mitochondrial aconitase. The super-conserved homology of the key IRE motifs in bold and highlighted are in yellow letters. (**C**) Maps of the 5′-UTR IRE stem-loops in the transcripts of APP, ferritin-H, and mitochondrial aconitase.

The IRE is present in UTRs of different mRNAs that produce proteins related to iron metabolism. IREs are constructed from basic RNA hairpins. Regulatory proteins use hairpins, the most prevalent RNA elements [39,40], as useful for binding [41]. The translation of IRE-containing mRNAs is controlled by IRPs’ differential recognition of IREs. IREs are around 30 nucleotide sequences found in the 5′ or 3′-UTRs of the mRNAs that encode iron metabolism proteins. They are folded into a hairpin with two short helices separated by a bulging C and a conserved six-nucleotide loop on top. Two forms of conserved information are present in every member of the IREs family, information particular to the IRE mRNA and information shared by all IRE mRNAs. A short (9–10 bp), double-stranded helix with an unpaired C at the center that causes a bulge is present in all IRE mRNAs (Figure 1A). Since all IREs create the mRNA A-helix with the identical bulge C and terminal loop sequence, the changes in IRE sequences across various mRNAs are quite minimal. A conserved C-G base pair across the IRE terminal loop in all IRE mRNA results in a bulge, an AGU, and a tri-loop [42]. A UGC/C or C bulge located five bases upstream of the terminal loop, which splits the hairpin stem into an upper and a lower stem, is present in IREs along with a highly conserved terminal loop.

The 5′-UTR of ferritin mRNA has one IRE. IRPs bind to the ferritin mRNA’s IRE and lower translation rates when the concentration of iron is low. In contrast, mRNA stability is enhanced when binding to several IREs in the transferrin receptor’s 3′-UTR. Conventionally, the IRE hexa-loop is numbered 14 through to 19, using ferritin IRE as an example. Between bases 14 and 18, there is a unique cross-loop base pair that gives the tri-loop appearance, together with the three nucleotides that come between them (15–17). As a result, the trailing 3′-base at position 19 is left hanging alone. The entire grouping of nucleotides 14–19 is referred to as a pseudo-tri-loop motif [43]. An IRE should have a CG base pair at position 14–18. IRE-containing mRNAs have been found in a variety of other proteins recently, including APP in Alzheimer’s disease [44,45], α-synuclein in Parkinson’s disease [46], and the α-hemoglobin stabilizing protein [47]. The RNA stem-loops encoded by the 5′-UTRs of transcripts associated with neurodegenerative diseases, namely those for APP, are depicted in Figure 1 panel A in comparison to ferritin, the general IRE mRNA. The pseudo-tri-loop is expected to be present in the secondary structure of APP IRE. The G7 residue is projected to be a bulge base in the APP IRE predicted structure, whereas the C8 residue is not [44]. A functional IRE is also encoded in the APP transcripts [45]. The canonical loop motif of the ferritin-H IRE stem-loop and the APP IRE are quite similar [45]. Base pairing in the APP IRE may produce a crucial AGA tri-loop [44]. Every mRNA encodes distinct variants of an IRE mRNA stem-loop at the 5′-UTRs that bind to IRP translational repressors. Ferritin IRE/IRP1 [48] and the APP IRE/IRP1 complex [49] seem to have comparable binding stabilities.

### 2.2. IRE Conservation

An IRE is a cis-acting mRNA regulatory motif with a distinct, highly conserved stem-loop sequence and structure [50]. The most prevalent mRNA elements are the IRE hairpins [40,51], which is a highly specific recognition element for the regulatory proteins (IRPs) [41]. In the apical loop, A15, G16, and U17 are the crucial contacts for identification. To maintain the high affinity binding to IRP, which is vital for regulatory function, they must be retained. Most of the IRE hydrogen bonds with IRP1 are formed by A15, G16, and U17 on the apex of the IRE pseudo-tri-loop motif. An appropriate IRE/IRP control requires these interactions. As a result, slots 15-16-17 should have an AGU triplet for an IRE. A weaker IRE/IRP interaction may arise if this apical loop is not A16G16U17; however, it might not support in vivo regulatory function. Mammals have a high degree of conservation in the structure and sequence of individual rings (IREs), which are typically composed of an apical loop motif and stem-loop element that are separated from a lower stem by a C-bulge [52,53,54,55]. Numerous IRE-like structures have now been hypothesized in different mRNAs, some of which encode proteins not only important in iron homeostasis but also not exclusively [56].

Figure 1 panel B illustrates the alignment of APP mRNA bulges and base pair differences in IRE helix base pairs of the 5′-UTRs in comparison to ferritin IRE [57] and mitochondrial aconitase IRE [48] mRNA transcripts. This homology includes the APP IRE, which differs from the established ferritin IRE but yet encodes a sequence resembling the IRE [58]. A 56% similarity between the ferritin and APP IRE sequences in this region is revealed by alignments. The predicted IRP1-binding AGU/AGA tri-loops, which have been demonstrated to be essential for IRP1 and IRP2 binding as well as translation suppression, and the CAGUGN loop domain of the canonical ferritin IRE are the focal points of this homology [44,59]. Varying mRNA stabilities [48,60,61] are a contributing factor to variations in the amount of proteins encoded in the mRNAs within cells. Quantitatively varied bindings of the IRP repressor or translation initiation factors, or even the Fe^2+^ signal itself, are the result of mRNA bulges and base pair variations in mRNA helix base pairs among members of the IRE mRNA family. Ferrous ion hexahydrate is the physiological iron signal. Different IRE structures result in different vivo iron responses. In vivo, the same amount of Fe^2+^ in the same tissue, like the liver, boosted ferritin protein production more than mitochondrial aconitase synthesis. Minor variations in the IRE mRNA structure among various IRE mRNAs explain this phenomenon.

Figure 1C illustrates the arrangement of the IRE-like mRNA stem-loop positions in APP, ferritin-H, and mitochondrial aconitase. At the 5′-UTR, the distance between the IRE and the mRNA cap and AUG start site can affect the regulation efficiency [57]. The distances between the IRE to start and IRE to cap can vary depending on the type of gene. Conversely, ferritin IRE differs far less from the IRE sequence variations in other mRNAs belonging to the same species, whereas the conservation of an individual IRE, such as APP IRE, is the same across species [62].

Similar to how iron controls the translation of ferritin mRNAs by IREs in their 5′-UTRs, iron also tightly controls the expression of the APP gene at the level of message translation. IRE mRNAs are bound by IRPs, which post-transcriptionally regulate ferritin and APP. Fe-mediated IRP/IRE binding to the 5′-UTR activity of ferritin and APP results in protein synthesis. When the protective APP and ferritin axis was lost, redox-active Fe^2+^ accumulated unregulated, causing neurotoxic oxidative stress. Neuronal cells treated with ferric ammonium citrate recently exhibited a rise in concentration and a time-dependent activation of ferritin-H and APP, iron storage proteins that help stabilize membrane-bound Fpn and can reduce intracellular hazardous redox-active Fe^2+^ concentration [63]. Iron chelation with desferrioxamine (DFO) appeared to be most effective in the presence of APP 3′and 5′-UTR sequences. IRPs are known to modulate intracellular iron homeostasis by controlling ferritin mRNA translation. However, IRP1 selectively binds to the APP IRE mRNA. Certainly, the APP mRNA translational control by iron and the APP gene transcriptional control by copper [64] each provide new genetic support for the model that APP is a metalloprotein expressed to detoxify metal controlled oxidative stress. Targeting the APP 5′-UTR will prove very useful to identify novel therapeutic interventions.

## 3. IRE/IRP Functional Interaction

**Figure 2:**
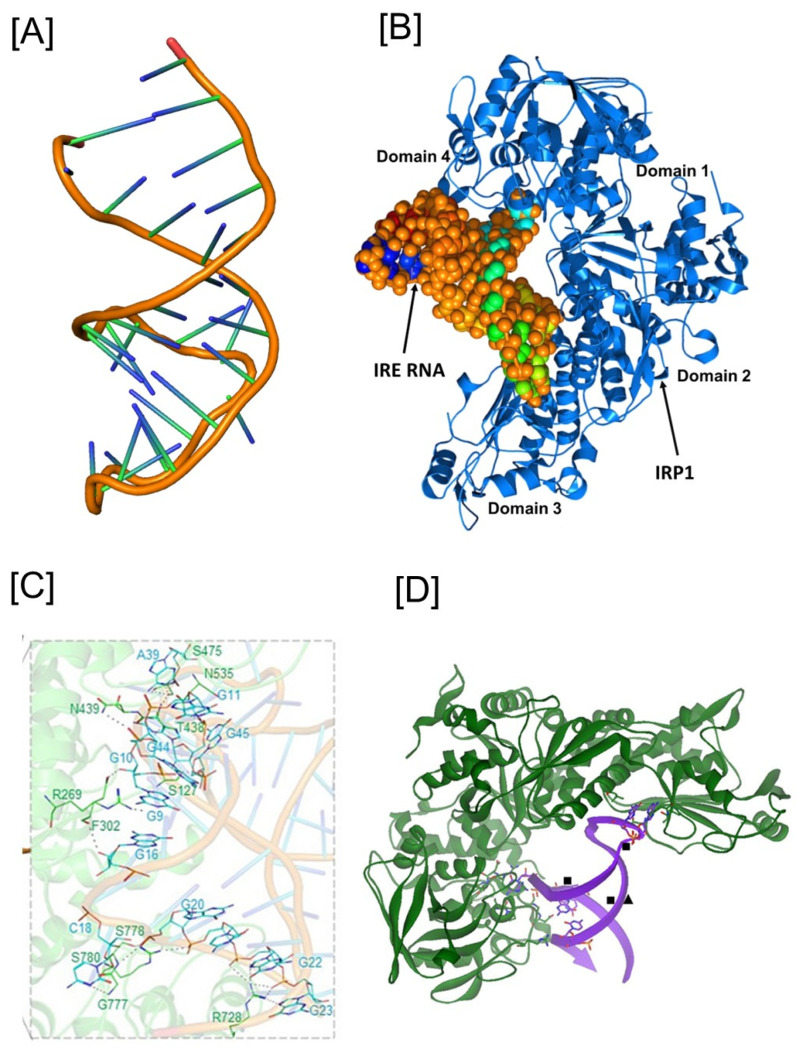
Structural models of the iron-responsive elements and its complex with IRP1 and Fe^2+^. (**A**) Tertiary structural models of the APP IRE mRNA, (**B**) Iron Regulatory Protein (IRP1) structural model in conjunction with IRE RNA [67], (**C**) close up of the IRE mRNA-IRP1 contacts at the RNA tri-loop and bulge (modification from ref [49]), (**D**) IRE mRNA sites influenced by metal binding related to the crystal structure of the IRE mRNA/IRP complex (modification of the figure originally published in ref [48]). (■), hydrated Mg^2+^, determined by solution NMR; (▲), Cu^1+^-1,10-phenanthroline, determined by RNA cleavage in O_2_.

When IRE interacts with IRPs, it recognizes the hairpin loop’s motifs and the stem’s bulge or loop. The precise binding mode of the IRE/IRP1 interaction of the mRNA in the IRP1 protein’s binding pocket is explored in Figure 2. The IRE mRNA is bent, and the IRP1 protein has an L shape. Between IRP1 protein domains 1–2 and 4, IRE is introduced into the complex. The IRE/IRP1 complex only has two contact sites that are widely apart in order to establish binding selectivity [42,65]. An AGU pseudo-tri-loop is produced by the C14-C18 base pair found in the apical loop of all IRE mRNAs, one of the two IRP interaction sites. Maintaining the orientation of the loop and the bulge for appropriate IRE/IRP binding is a crucial function of both the lower stem of the IRE, located below the bulge, and the upper stem of the IRE, situated between the terminal loop and the bulge. A15 and G16 flip out of the helix and enter the IRP structure deeply into the IRE/IRP complex [42]. The structural conformation of IRE mRNA to bind to a protein molecule determines its functional ability [66]. The IRE mRNA tertiary structural model, IRE complex with IRP1, and interaction between nucleotide bases and amino acid residues are shown in Figure 2A–C [49,67]. Conformational modifications in the mRNA and probably the unliganded protein are necessary to explain the discrepancies between the solution structures of free mRNA and protein-bound IRE mRNA. A significant portion of the IRE surface is open to interactions with proteins and metal ions in the RNA/protein crystal structure [68]. It seems likely that IRP1 uses the same bonding strategy to bind all naturally occurring IRE mRNA. The structure of the IRE bound to IRP1 is different from the predominant structure of the mRNA in solution, highlighting the significance of conformational flexibility for this high affinity binding and suggesting that the conformational flexibility of the IRE mRNA may play a role in the affinity differences observed between these IRE/IRP protein bindings. There may be less IRE/IRP binding if this apical loop is not A15 G16 U17; however, it might not provide in vivo regulatory function.

All IRE configurations can be recognized by the regulatory protein IRP [48,59]. Subtle, conserved changes in the IRE structure and sequencing result in a variety of RNA–protein complexes. Each member of the IRE family has a somewhat different RNA–protein complex binding stability. The alterations in IRE/IRP stability have physiological ramifications. For example, under the same conditions, ferritin and mitochondrial aconitase IRE/IRP have a ten-fold difference in stability [48], while APP and ferritin showed a two-fold difference in stability for IRE/IRP binding in a solution [49,69]. Variations in the quantities of cellular proteins encoded in mRNAs are a result of variations in IRE stabilities. Compared to APP and mitochondrial aconitase IREs, ferritin IRE and IRP create a far more stable complex. As a result, ferritin mRNA translation is more robust than the other mRNAs, which collectively comprise the less stable IRE/IRP complex, to slight variations in intracellular iron levels. From a physiological perspective, the structural differences between ferritin, mitochondrial aconitase, and APP IRE correspond to functional variations in the cell metabolism of each protein encoded in an mRNA. This is because mitochondrial aconitase is continuously needed for bioenergetic cellular activities, while ferritin and APP are occasionally needed for their iron-concentrating activities. Because IRP-binding affinities for each unique IREs are quantitatively varied, the fraction of mRNA inactivated by IRP binding will differ for each IRE mRNA at any given time [70].

APP IRE binds to IRP1 similarly to how ferritin IRE binds to IRP1. The IRP1-binding site’s functionally active residues were used by APP IRE to bind IRP1 in a cleft. APP IRE was able to firmly fit into the binding pocket of the IRP1 protein due to its structural complementarity [49]. Numerous bonds hold the APP IRE and IRP1 complex together. A pocket produced in domain 3 at a region restricted by domains 1 and 2 in the globular form was the site of eleven hydrogen bonding between the stem-loop of APP IRE and the amino acid residues of IRP1 (Figure 2C). Additional interactions are formed between the IRE stem’s nucleotide bases and amino acid residues in domain 4 of IRP1. In the IRE/IRP complex, the APP IRE flips out its terminal loop bases, and a sudden mid-helix turn deforms the IRE backbone. It is anticipated that the pseudo-tri-loop and the conserved C8 residue will both be present in the secondary structure of APP IRE mRNA. G5 is anticipated to be a bulge base; however, the C8 residue is not predicted to be a bulge base in the APP IRE-projected structure [44,49]. The largest variations in IRP1 binding in solution occur in the in vivo response to iron levels, where there is at least an order of magnitude difference between APP and ferritin mRNA [49,70].

## 4. Fe^2+^ Sensing IRE mRNA to Influence Protein Binding

Natural differences in the helix base pairs of IREs carrying mRNA coding for different proteins are associated with quantitative changes in IRP1-binding affinity and the magnitude of the iron response in vivo. The IRP/IRE mechanism, which post-transcriptionally alters target gene expression in accordance with cellular iron levels, provides the crucial regulation of iron homeostasis at the cellular level. The IRE mRNA-based ethidium bromide displacement, impacts on NMR spectra, binding of metal complexes, and the lack of expected metal ion binding sites on IRP beyond the [4Fe-4S] cluster insertion site have all been shown to be directly impacted by metal ions, according to studies [48,71]. Furthermore, several RNA/protein connections have locations that are extremely vulnerable to Fe^2+^-EDTA breakage [58], indicating interactions with the solvent, Fe^2+^-EDTA, or both. The notion that the IRE mRNA mid-helix bulge serves as the binding location of regulatory metal ions is supported by previous research on Cu-1,10-phenanthroline and modeling of Co (III)hexamine binding [72]. Bulge bases are locations in other RNAs where metal ions can bind [73]. A significant portion of the IRE mRNA’s surface is available for interaction with other biomolecules, including metal ions, in the RNA/protein crystal structure [42]. According to the data, the IRE mRNA Fe^2+^ binding site is located close to the stem-loop bulge that forms AGU and C (Figure 2D). Fe^2+^ binding to IRE mRNA alters the mRNA structure, according to experiments using IRE mRNA and the fluorescent reporter 2-aminopurine [74]. Fe^2+^ is the physiological signal that targets the IRE mRNA to weaken IRP binding and promote mRNA translation, as evidenced by its significantly greater metal selectivity on IRP binding to IRE mRNAs than other metal ions like Mg^2+^ and Mn^2+^ [48].

**Figure 3:**
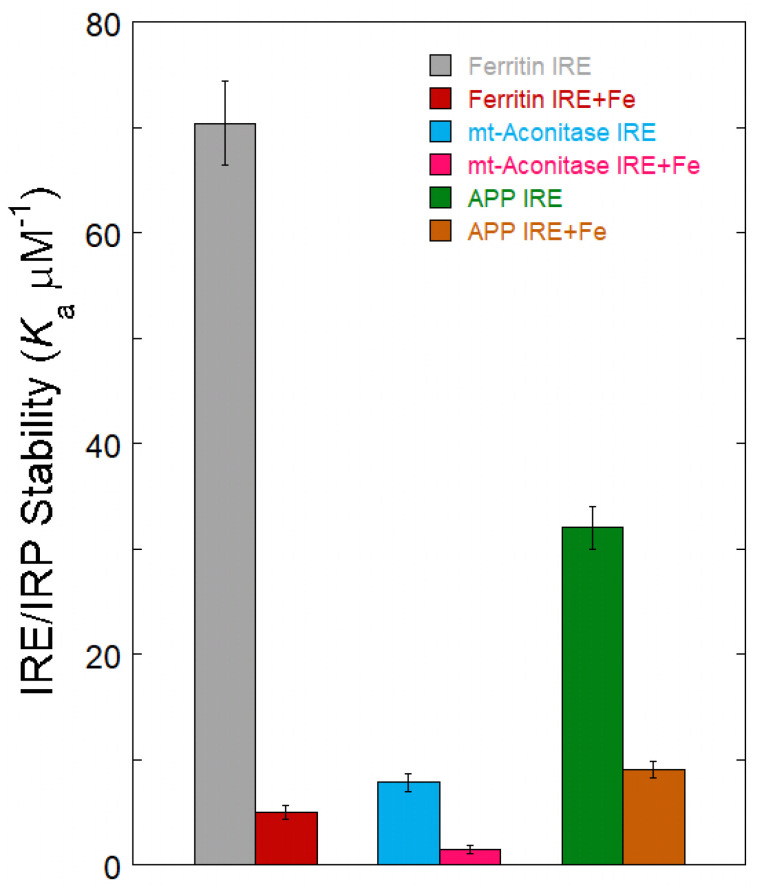
Fe^2+^ influences the stability of the APP, ferritin, and mitochondrial aconitase IRE/IRP1 complex formation. Data for the preparation of the figure were taken from ref. [48,49].

The molecular mechanism was unknown until the recent discovery that iron destabilized APP IRE/IRP1 complexes [49]. Fe^2+^ interacts directly with IRE mRNA to reduce the binding affinity of IRE/IRP in solution. The biggest variations in IRP1-binding affinity in solution are shown in ferritin, mitochondrial aconitase, and APP IRE mRNA, which respond to iron levels in vivo by at least an order of magnitude (Figure 3) [48,59]. Over the same concentration range, ferritin IRE/IRP repressor complex stability is significantly reduced by Fe^2+^ in comparison to mitochondrial aconitase and APP IRE. This suggests that variations in IRE mRNA structures have a role in the variations in Fe^2+^ responses seen in vivo. According to these results, the IRE/IRP repressor complex specifically reacts to Fe^2+^ for every IRE mRNA. Numerous links between the IRE mRNA and IRP protein complex provide stability, which is competitive with the Fe^2+^ destabilization of the IRE/IRP complex. Fe^2+^ binding to the exposed locations on the IRE mRNA is most likely the cause of the observed instability of the IRE/IRP complex (Figure 2D). The stability variations of the IRE/IRP complexes reflect structural differences among ferritin, mitochondrial aconitase, and APP IREs. The physiological role of each encoded protein was likewise reflected in the three IRE mRNAs’ distinct iron responses. A combinatorial array of IRE/IRP complexes with a variety of physical stabilities that are adjustable by the Fe^2+^ signal is produced by the family of IRE mRNA structures that are selectively recognized by IRP.

The repressor protein IRP is released from IRE mRNA as a result of the conformational changes brought on by Fe^2+^ binding. On the other hand, to boost translation, Fe^2+^-induced RNA conformational changes to improve binding to the eukaryotic initiation factor (eIF)4F. IRP, an IRE-specific regulatory, and eIF4F, a translation factor protein that binds to all mRNAs, are the two recognized protein types that regulate the activity of IRE mRNA. Fe^2+^, on the other hand, makes the IRE/eIF4F complex more stable [74]. Fe^2+^ ions then drive the binding competition away from IRP and toward eIF4F as they compete for IRE binding. Two mRNA–protein interactions are affected in opposing ways when Fe^2+^ binds to IRE. Repressor IRP separates from mRNA, and the mRNA is transcribed into proteins such ferritin (stores iron), APP (transports iron in brain), DMT1 (iron uptake protein), and Fpn (exports iron). Because eIF4F sites are accessible on IRE mRNAs during IRP dissociation, eIF4F binds with IRE and recruits ribosomes to mRNA, which explains ancient observations that high iron in cells and animals induces mRNAs to transfer from cell supernatant fractions (free mRNA) to polyribosomes (bound mRNA), increasing ferritin synthesis up to a hundred-fold. The process relies on the direct interactions between Fe^2+^ and IRE mRNAs as well as associated conformational changes in IRE mRNA that reduce the likelihood of IRP binding [74].

Numerous findings demonstrated the specificity of the interaction between IRP1 and APP IRE [44,49]. Similar to the mechanism by which iron regulates the translation of ferritin mRNAs by the IRE stem-loop in their 5′-UTR, IRP1 can extract APP IRE mRNA from cellular quantities of free Fe^2+^, enabling eIF4F and ribosome assembly to translate the APP protein. Similar to ferritin IRE, the APP IRE stem-loop bound to IRP1 with significant affinity. In response to intracellular iron levels, this tight APP IRE/IRP association was equally noticeable in human diseased brain lysates, SH-SY5Y cells, and H4 neuroglioma cells [44]. It has been suggested that a pathogenic characteristic of the AD brain that has not yet been identified is reflected in the changed binding of IRP1 to the ferritin IRE.

## 5. Fe^2+^-Induced APP mRNA Translation

Iron overload has a role in the development of AD by causing amyloid protein overexpression, Aβ buildup, and NFT formation. The existence of an IRE in the 5′-UTR of the APP mRNA transcript has been used to establish a direct connection between Fe^2+^ homeostasis and the etiology of AD [45,57]. In a manner that regulates the iron-dependent control of intracellular APP production, IRE is selectively responsive to intracellular iron levels. Fe^2+^ levels have been demonstrated to influence the translation of APP mRNA in astrocytes and neuroblastoma cells by a mechanism like iron control of ferritin mRNA translation [45,75]. The signaling cascade of IRPs and IREs modifies the cellular iron balance at the translational level [76]. mRNA-binding proteins like IRPs are known to regulate translation through their inducible interactions with IRE. Iron can have opposing effects on distinct IRE RNAs due to the two distinct positions of IREs in mRNA. When IREs are present in the 5′-UTR, it controls the binding of ribosomes and initiation factors to promote mRNA translation. Conversely, when the IRE is present in the 3′-UTR, it controls target mRNA nuclease binding and mRNA breakdown [62,77].

Although IRP binding to 5′-UTR is known to restrict mRNA translation and to limit ribosome binding, the specific stages involved in building the translation initiation factors’ complex that are hindered are still unknown. Target mRNA in iron-deficient cells can be shielded from endonuclease cleavage by IRP binding to an IRE at the 3′-UTR of transcripts [77]. Thus, the interaction of IRPs with IRE can increase target mRNA translation and prolong the half-life of transcripts. In contrast, an endonuclease attack and degradation brought on by IRP’s dissociation from an IRE at the 3′-UTR reduce the likelihood that target transcripts in iron-depleted cells will be translated. When iron overload occurs, the IRP/IRE interaction can both destabilize transferrin receptor (TfR) mRNA and disrupt and enhance ferritin transcript translation [77]. Consequently, in conditions of iron overload, iron export and storage might be augmented while iron absorption is restricted [78]. It was not known until recently how cellular iron signals alter IRP’s affinity for APP IRE [49].

Furthermore, the process of binding ribosomes is quite intricate and involves the binding of numerous initiation factors and proteins to form an initiation complex that includes ribosomal subunits, initiator tRNA, and mRNA. It is still unknown exactly what happens in what order IRP is released, and an initiation complex with an IRE mRNA is put together. Because there is little distance between the start of the mRNA and the IRE, several characteristics imply that IRE structures work in concert with other components of the mRNA structure [57]. The initiator AUG is embedded in the IRE of some mRNAs, such as mitochondrial aconitase; the functional significance of initiation at the IRE is unknown. To control the stability of a protein repressor complex that prevents ribosome binding and protein synthesis, these IRE structures bind iron preferentially. Binding eIF4F to IRE results in positive regulation. The common IRE mRNA loop and bulge that comprise the distinct protein binding sites for each IRE are used to characterize the IRE mRNA. Changes in the base pairs of the individual IRE helix alter the binding of metabolites (Fe^2+^) and repressor proteins (IRPs), converting protein production in vivo environmental iron. Ferritin protein synthesis consumes the iron signal, creating a regulatory feedback loop with iron. Consequently, ferritin protein reduces the amount of free cellular iron, raises the binding of IRP1 to ferritin IRE mRNA, and lowers the rates of ferritin protein synthesis.

**Figure 4:**
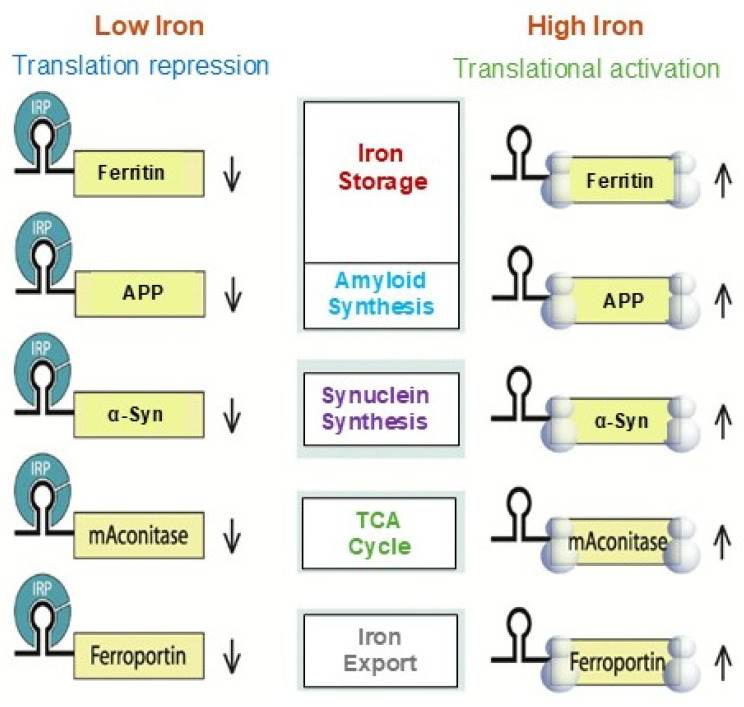
A model that illustrates how binding of IRE RNA to IRP causes iron to stimulate the translation of iron regulatory proteins. Protein expression and depression according to cellular iron levels (high or low). This figure was amended from reference [48].

Figure 4 represents the detailed Fe^2+^-induced translation regulation of the IRP/IRE system. IRPs could both promote and inhibit translation [62,77]. These systems control iron homeostasis at several levels, resulting in fine-tuning of iron transport, storage, and regulation at the cellular and systemic levels in vivo. However, an excess of iron in the brain’s tissues causes neuronal death when the iron regulating system is out of balance due to genetic mutations and/or diseases, affecting iron-balancing proteins such as ferritin, ceruloplasmin (CP), APP, TfR, DMT1, and Fpn [79]. Raising the quantity of iron within cells can modify not just the shape of IRE mRNA, which in turn affects IRP and eIF4F binding, but also the protein itself.

**Figure 5:**
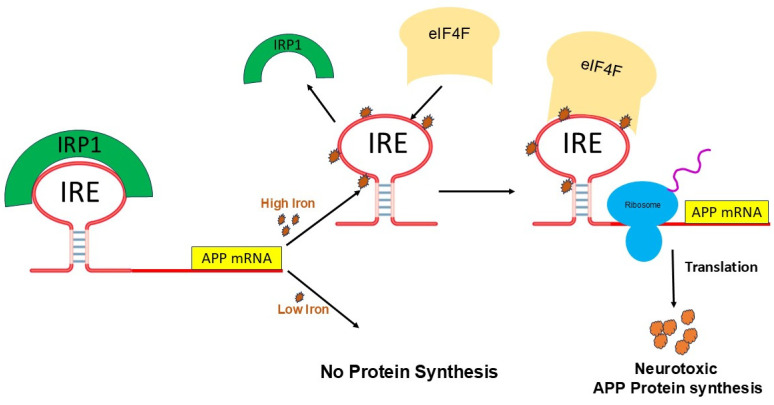
A possible mechanism of action for the molecular model explaining how elevated iron levels prevent IRP binding and encourage eIF4F and ribosome binding to start the translation of the neurotoxic protein by APP IRE RNA in the brain, hence contributing to Alzheimer’s disease.

Iron homeostasis will be hampered by the disruption of the IRP/IRE signaling system, which may have a role in the overexpression of the amyloid protein and progression of AD pathogenesis. The physiological iron signal on IRE mRNA translation is represented by a proposed model (Figure 5) for iron-regulated neurotoxic amyloid protein production. For iron-induced protein biosynthesis to occur, IRE mRNA must sequentially interact with IRP1, iron, eIFs, rRNA/protein complexes with ribosomes, and tRNA/protein elongation factor complexes. IRP1 binds significantly to the IRE mRNA suppression of neurotoxic protein synthesis at low cellular iron levels, where ribosome binding and the eIF4F are inhibited. On the other hand, iron can also directly attach to the target IREs at high cellular iron levels, changing the mRNA’s conformation. The detachment of IRPs from the target mRNA is encouraged by the structural changes in mRNA caused by iron binding. This facilitates the interaction between eIF4F and IRE mRNA and speeds up the translation of the neurotoxic amyloid overproduction that causes Alzheimer’s disease. The regulation of APP expressions by iron can be derived from the genetic regulation of ferritin mRNA translation and TfR mRNA stability. By focusing on and blocking the translation of APP gene expression without affecting general translation, this information will be pertinent to RNA-directed therapeutic targets that aim to cure AD [80].

## 6. Brain Iron Transport and Regulation

Iron is the most abundant and essential element in the brain as well as the whole body. Two oxidation states make up the majority of free iron under physiological conditions: +2, or divalent ferrous Fe^2+^, which is somewhat soluble, and +3, or trivalent ferric Fe^3+^, which is extremely insoluble. Because of its exceptional dual functionality as an electron acceptor and donor, iron plays a crucial role in cellular metabolism, including oxygen transport and the mitochondrial electron transport chain. The cytosol’s free Fe^2+^ makes up a labile iron pool (LIP ~3 μM) that cells can use. Additionally, if it is not used right away, cytosolic ferritin can quickly sequester it into a non-reactive state. Because ferritin complex protein nanocages move cytoplasmic Fe^2+^ through intra-cage iron channels to cage-embedded enzyme (2Fe^2+^/O_2_ oxidoreductase) sites where ferritin biomineralization is initiated, they generate integrated iron-oxy biominerals (Fe_2_O_3_•H_2_O). The concentration of iron in the LIP may rise detrimentally and cause oxidative damage and cell death if iron concentrations surpass the cellular iron sequestration capacity of storage proteins [81].

Iron metabolism-related mRNA transcriptional levels primarily control iron homeostasis in nerve cells. The primary proteins implicated in iron metabolism in the brain are ferritin, Tf, TfR1, Fpn, DMT1, and IRPs [82]. Through the process of endocytosis, iron– transferrin complexes create endosomes inside cells. The iron and Tf/TfR complexes dissociate as a result of the proton pump’s impact on the endosome membrane, which also causes the endosome’s pH to drop to an acidic level, converting Fe^3+^ to Fe^2+^ [83] and exporting into the cytosol, increasing the LIP in neurons. DMT1 releases iron from endosomes in the cytoplasm of brain capillary endothelial cells, which is then exported into the brain by Fpn [84,85]. In pH 7.4, Tf dissociates from TfR and re-enters the blood. TfR1-mediated endocytosis allows neurons to absorb transferrin, the main iron transport protein in the brain, after it has been absorbed in endosomes. Iron export from neurons is carried out by Fpn and circulating CP, and Fpn requires the amyloid precursor protein, which is transported to the membrane by the microtubule-associated protein tau, to stabilize it. The cytosolic or membrane-bound ferroxidase, like CP, is usually associated with the export of Fe^2+^ from cells. Prior to dispersing across the extracellular environment, it oxidizes reactive Fe^2+^ to its less reactive Fe^3+^ state. Tf has the ability to bind Fe^3+^ in the extracellular space. Excess intra-lysosomal or cytosolic iron can result in the production of free radical oxidants. Microglia have been shown to absorb iron and export ferritin, although the underlying processes are unclear. For oligodendrocytes to generate myelin and create the myelin sheath that surrounds neurons, iron is also required.

By regulating the binding of IRPs to IRE, iron maintains intracellular iron homeostasis and controls the transcription of iron-related proteins [57]. Additional interlocking mechanisms that primarily control intracellular iron homeostasis are hepcidin-ferroportin-mediated serum iron level regulation and hypoxia inducible factor-2α mediated transcription [17]. Fpn1 is the major receptor for hepcidin. Hepcidin and Fpn1 interact directly to control iron homeostasis. This connection includes the internalization and degradation of Fpn1, which decreases cells’ capacity to export iron and raises the risk of intracellular iron overload. Hepcidin decreased the expression of TfR and DMT1 in astrocytes and neurons in addition to downregulating the expression of Fpn1. Studies have demonstrated that APP is a regulator for iron homeostasis, which can interact with Fpn1 to regulate the efflux of ferrous ions [86]. Fpn controls intracellular levels of excess Fe^2+^ and exports it to the extracellular environment because it damages neurons and produces ROS. Thus, there is careful regulation of both the level of iron and the ratio of Fe^2+^ to Fe^3+^. Tau acts as an intracellular microtubule-associated protein, which can transport the produced APP to the cell surface to promote iron output [87].

## 7. Role of Iron in the Occurrence of Alzheimer’s Disease

**Figure 6:**
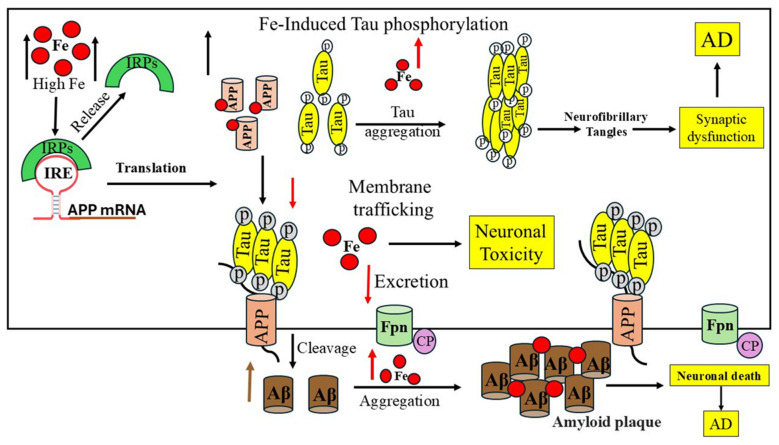
Alzheimer’s disease and dysregulation in brain iron homeostasis. A model that illustrates how alterations in reaction to elevated cellular iron levels in both healthy and Alzheimer’s disease patients cause iron-induced translation of amyloid plaque by APP IRE RNA. This figure is modified from reference [96].

AD brains exhibit an excess iron accumulation, as evidenced by changes in the expression of most iron-regulating proteins, and most amyloid plaques contain iron and ferritin-rich cells [88,89]. AD neuropathology is exacerbated by the dysregulation of the redox-active iron. AD-related NFTs and insoluble Aβ-plaques contain high levels of iron. Fe^2+^ promotes Aβ neurotoxicity by producing free radical damage and oxidative stress in the brain areas affected by neurodegeneration in AD [17]. Fe^2+^ encourages the aggregation and oligomerization of Aβ-peptides, and the interaction between iron and Aβ peptides is harmful [90]. Aβ’s strong affinity for binding iron and its ability to chemically reduce metals, which results in the generation of catalytic hydrogen peroxide and consequent oxidative damage, are the real causes of the oxidative damage linked to it [91]. Iron-induced oxidative stress may also promote the hyperphosphorylation and aggregation of tau [92,93]. With an emphasis on the iron’s capacity to bind to the Aβ-peptide and enhance Aβ toxicity, Figure 6 depicts the process of brain iron dysregulation and its connection to AD. Hyperphosphorylation and tau aggregation hinder the transport of APP to the cell membrane by reducing the amount of soluble tau, which increases the development of LIP in neurons [89,94,95]. 

Another significant pathogenic characteristic of AD is NFTs, which are primarily composed of the phosphorylated tau protein. Iron accumulation in neurons with NFTs has been documented [4,97]. In addition to Aβ-peptides, iron can bind to the tau protein and cause tau protein phosphorylation and hyperphosphorylation, which results in aggregates of hyperphosphorylated tau protein. The aggregation of the hyperphosphorylated tau protein caused by Fe3+ can be reversed by reducing Fe^3+^ to Fe^2+^ [98] and/or iron chelators [99]. These findings suggested that the accumulation of the hyperphosphorylated tau protein to produce NFTs may be significantly influenced by iron. Additionally, it has been demonstrated that the tau protein has an indirect role in the pathophysiology of AD by aiding in the transport of iron ions in brain neurons [100]. Additionally, both in vitro and in vivo research has demonstrated that iron contributes to tau protein hyperphosphorylation by activating the glycogen synthase kinase-3β (GSK-3β) and cyclin-dependent kinase (CDK5)/P25 complex [101].

Aβ oligomers, which are toxic forms of Aβ, have been found to be accelerated and stabilized by the Fe^2+^ ion. Fe^2+^ and Fe^3+^ interact with APP and Aβ to promote Aβ aggregation into fibrous forms, according to previous studies. The form of amyloid is altered by Fe^2+^’s interaction with the Aβ protein’s amino acids. Neuronal mortality is accelerated when β-secretase cleaves monomer Aβ_1–42_ into more lethal Aβ oligomers because Fe^3+^ coupled to Aβ is readily converted to Fe^2+^ and enhances ROS generation. Aβ can exacerbate iron overload and AD by impairing mitochondrial function, converting Fe^3+^ to Fe^2+^ with redox activity, and causing oxidative stress [102]. Additionally, it has been demonstrated that in cultivated SHSY5Y cells, iron exposure stimulates APP aggregation [103]. According to a different study, Aβ can also dramatically lower iron’s redox capacity, which may suggest neuroprotection and metal chelation of Aβ during the pathophysiology of AD. However, in some situations, this might become hazardous [93].

Iron mis-regulation and AD pathogenesis are closely related because cellular iron levels directly control APP translation through the IREs found in the 5′-UTR mRNA, making APP a metalloprotein [45]. Normally, the metalloprotease α-secretase cleaves most neuronal APP (in a non-amyloidogenic pathway) to produce neuroprotective APPs that are released from cells into the bloodstream, cortex, and cerebrospinal fluid (CSF) [1]. The amyloidogenic pathway (neurodegenerative) produces Aβ-peptides by first cleaving APP with β-secretase and then γ-secretase. Aβ production and accumulation in the brain are inhibited by activating the α secretase pathway. Higher iron concentrations in neurons increase the amount of APP protein expression at the translational level that can undergo amyloidogenic processing and enhance the generation of Aβ. Furin regulates the proteolytic activation of the dormant forms of α-secretase and β-secretase [104]. Iron overload reduced furin activity because iron levels in cells affect furin transcription, which lowers furin protein concentrations and favors β-secretase activity, the amyloidogenic pathway, and the generation of Aβ. Additionally, it has been shown that APP stabilizes surface Fpn, which promotes iron efflux from neurons [63,105].

Iron influences how APP is processed; at high concentrations, iron prevents APP from maturing while leaving immature APP intact. APP is the one that undergoes proteolysis to yield the Aβ-peptide, which then combines to form the primary building block of amyloid plaques [27]. Immature APP translational expression causes poor iron metabolism and increased neurotoxic oxidative stress, which in turn causes Fe^2+^-induced neurotoxicity. Because tau deficiency inhibits APP trafficking to the membrane, it cannot interact with Fpn and inhibits iron efflux from neurons, which results in intracellular iron accumulation and dopaminergic neuronal death, dementia, and AD.

It is recognized that a number of hereditary iron metabolism disorders in humans, including AD, PD, and Hallervorden–Spatz syndrome, cause neurodegeneration and brain iron imbalance [106]. Numerous neurodegenerative illnesses, including neurodegeneration with brain iron accumulation (NBIA), have been shown to have abnormally elevated iron concentrations in the brain. It has been shown that anomalies in iron metabolism, primarily in neurons or astrocytes, are the direct cause of neuroferritinopathy and ceruloplasminemia, which are brought on by mutations in the ferritin-L chain and CP [107,108]. Moreover, the most prevalent type of NBIA is pantothenate kinase-associated neurodegeneration (PKAN). Neuronal death is believed to be directly caused by cysteine-mediated iron buildup in the brain, which is brought on by mutations in PANK2 through the free radical pathway [109]. Additional evidence has demonstrated that certain genes involved in lipid metabolism (PLA2G6, C19orf12, and FA2H) are necessary for ferroptosis, an iron-dependent cell death, and that genes primarily engaged in autophagy (WDR45, ATP13A2) are essential for cellular iron management. These results imply that lipid and autophagy metabolisms are linked to iron metabolism [110]. It has been discovered that APOE4 increases the risk of AD via regulating ferritin, an iron homeostasis protein, and inversely regulating the effects of iron on brain function [111]. The report indicates that iron build-up and elevated iron concentrations (~1 mM) in the vicinity of amyloid plaques and NFTs in AD patients [112] have a substantial association with tau pathology and Aβ-plaque pathology [112].

## 8. Small Molecule Therapeutic Targeting mRNA for AD

Small molecule-based Aβ aggregation modulation has been shown to be a very effective strategy [113]. Current RNA drug development relies on the secondary structure of RNA, while the 3D structure is a more typical pharmacological target for proteins. The benefit of RNA therapeutics is that their target size is smaller than that of proteins. Neurodegeneration in AD and PD is likely caused by the regulation of APP and α-synuclein translation, which has been linked to the IRE/IRP signaling pathway. Therefore, finding small-molecule IRE-targeted inhibitors to lower APP levels and prevent protein aggregation may be useful for treating AD. JTR-009, paroxetine, posiphen, phenserine, carvediol, yohimbine, and other interesting small compounds are currently being explored in several therapeutic trials for AD and PD. These drugs bind to a specific location in the IRE mRNA in solution, changing mRNA function [114,115]. These findings demonstrated that the tiny RNA-binding molecules have the same selectivity in solutions as they do in living cells, binding to folded target RNA structures. In one instance, JTR-009, a benzimidazole, was found to limit amyloid burden in mouse models of AD by reducing the production of toxic Aβ in SH-SY5Y neuronal cells more than other well-tolerated APP 5′-UTR directed translation blockers, such as posiphen and phenserine [115]. These small compounds maintained IRP1’s connection with ferritin IRE mRNA while blocking IRP1’s ability to bind to the APP mRNA. These investigations offer crucial information for creating tiny compounds that specifically lower the generation of Aβ and APP in the brain in AD. These medications directed via the APP 5′-UTR represent a novel approach to find RNA-based medicines that reduce APP translation and Aβ-peptide production for AD treatments.

To evaluate their anti-amyloid potential in the transgenic TgCRND8 AD mouse model, other strong IRE inhibitors such as paroxetine, N-acetyl cysteine, an antioxidant and iron chelator, and erythromycin were used [80]. Research revealed that paroxetine affects the 5′-UTR of the APP transcript, which in turn affects APP expression [116]. Additionally, medications including paroxetine and DFO, as well as a number of new substances, have been shown to inhibit aberrant metal-promoted Aβ buildup. A subset of these treatments work by modulating APP translation and cleavage through APP 5′-UTR-dependent processes, resulting in the non-toxic sAPPα. Their action has been tested as bonafide anti-amyloid and anti-synuclein agents for the treatment of AD and PD [117,118]. In addition to these drugs, AF102B is a neuroprotective m-1 muscarinic agonist that is used to treat Sjogren’s syndrome. It also has the ability to trigger the release of APP(s) by activating the nonamyloidogenic pathway of APP expression (α-secretase). Additionally, this medicine can be examined for its ability to improve APP–Fpn complexes to prevent ROS formation and efflux toxically embargoed iron. Since Fe causes APP to spread, RNA-based small-molecule inhibitors that target and regulate APP mRNA expression offer yet another class of therapeutic medicines that may be used to treat amyloid toxicity in AD.

A lack of enough specificity and correct translational models, loss of Aβ physiological homeostasis, and inability to deliver during the optimal therapeutic window have most contributed to the failure of the Aβ-targeting pharmacological studies. The phase III studies have recently failed on a variety of targets, including β-secretase, γ-secretase inhibitors (such as semagecestat, avagacestat, and tarenflurbil), monoclonal antibodies specific to Aβ (solanezumab), crenezumab, gantenerumab, and some tau aggregation inhibitors. The phase 2 clinical trial for the anti-tau medication donanemab, which aims to prevent the accumulation of tau tangles, failed in August 2024; fosgonimeton, which aims to improve brain function by focusing on neurotrophic pathways, failed in September 2024; and dalzanemdor, which was tested on patients with mild cognitive impairment and early-stage dementia, failed to demonstrate any discernible improvement in AD and was discontinued in October 2024.

Drugs such as aducanumab, donanemab, and lecanemab have been approved by the FDA to reduce Aβ plaques. These drugs provide strong evidence that removing amyloid from the brain can slow down AD, and the amyloid hypothesis, whose potential had been uncertain after dozens of drug failures, has disease-modifying potential. Together, these breakthroughs highlight a dynamic shift in AD, from improved diagnostics to more accessible treatment and novel therapeutics approaches.

Another intriguing method for treating AD is the use of traditional Chinese herbal remedies like the Bushen Tiansui Formula [119] and Lonicerae Japonicase Flos [120]. Additionally, Apelin-13, a prominent neuropeptide that inhibits inflammation, has positive effects on memory, cognition, and neuronal damage. Additionally, apelin-13 attenuates inflammation to regulate the BDNF/TrkB pathway against cognitive loss in a SIT-induced rate model of sporadic AD [121]. Moreover, quercetin, a bioactive substance that is a member of the flavonoid class, has promising neuroprotective and anti-inflammatory properties in the treatment of AD. However, issues including low bioavailability and limited permeability across the blood–brain barrier (BBB) limit its therapeutic potential. Quercetin-functionalized nanoparticles are now a novel treatment agent for AD to advancements in nanotechnology [122]. Furthermore, Aβ is thought to be the main treatment target for AD. Eliminating Aβ alone, however, has shown limited therapeutic efficacy in AD patients, potentially ignoring the metabolic problems brought on by AD, such as insulin resistance. The dysregulation of GSK-3β in aging is evident [123].

Guanidinium-modified cyclodextrin and calixarene coassembly as insulin delivery systems address this problem. This strategy looked beyond the traditional method of removing Aβ in order to address the metabolic abnormalities characteristic of AD in a novel way [124]. Furthermore, ROS-related nanoparticles for AD treatment [125]; nanotechnology-assisted medication delivery targeting AD [126]; and bioactive compound-fortified nanocarriers for neurodegenerative disease treatment [127] are methods that appear promising as a treatment option for AD.

## 9. Metal Chelator Therapeutics in AD

It has been observed that metal ions are essential for the stabilization and acceleration of Aβ oligomers. Thus, utilizing metal chelators to target metal ions is a possible therapeutic approach to reduce Aβ toxicity. Oxidative stress brought on by excessive iron deposits in the brain can cause protein aggregation and neuronal death [128]. As a result, BBB-penetrating iron chelators ought to be useful in treating AD patients.

The metal ion hypothesis is a widely accepted mechanism that plays a significant role in the neuropathogenesis of AD. Strong involvement in Aβ aggregation and its toxicity is indicated by the large amounts of metal ions, such as Fe^2+^, Cu^2+^, and Zn^2+^, that are coordinated with the Aβ peptide in senile plaques [129]. When metal binds to Aβ, it stabilizes the toxic oligomeric state, which produces ROS and breaks down synapses, ultimately resulting in the death of neuronal cells [130]. Aβ disrupts metal ion homeostasis in the brain by sequestering physiologically significant metal ions [131]. Metal chelators have been used to disrupt Aβ–metal interactions in an attempt to restore metal ion homeostasis in the brain and lessen the neurotoxicity caused by the Aβ–metal complex. DFO was the first metal chelator to dissolve metal-induced Aβ aggregates and can decrease dementia in AD patients [128]. Its weak BBB permeability, rapid in vivo breakdown, and other negative side effects, however, limited its use. For AD, metal chelation is one of the treatment options [132]. It was shown that APP mRNA encodes new, fully functional iron-responsive regions that regulate APP translation. By preventing APP mRNA translation from its 5′-UTR, DFO, a highly selective iron chelator, reduced intracellular APP levels [133]. Accordingly, intracellular iron chelation reduced intracellular APP expression by increasing IRP1’s binding to the APP IRE [44].

Clioquinol (CQ), an iron–copper–zinc chelator, has been shown to have broad specificity in reducing plaque burden by dissolving extracellular fibrillar Aβ. Its ability to penetrate the blood-brain barrier can also reduce plasma Aβ levels and clinically mitigate cognitive decline in AD patients [27,134]. Nonetheless, myelinopathies have been linked to clioquinol [135]. Moreover, in a variety of in vivo scenarios, iron chelators such as deferiprone, DFO, deferasirox, D-607, and VAR10303 can all considerably reduce neuronal loss [136,137].

Iron-chelating nanogels with DFO components may be more successful than DFO alone at treating iron overload, according to a recent study; further research on these nanogels is necessary [138]. The clinical effectiveness and safety of iron-chelating techniques in AD treatments will likely be further established by thorough future research. The elevation of iron in AD patients’ brains is the focus of another intriguing strategy for metal-based treatments.

## 10. Conclusions

Recent evidence on APP mRNA, IRP and iron signaling pathway in brain iron metabolism suggests a pathological and therapeutic link in AD. Interestingly, many reports relevant to the brain iron overload, Aβ metabolic disorders, tau protein hyperphosphorylation, oxidative stress and gene mutations are potential pathophysiological factors associated with AD. It is still not clear whether in AD pathogenesis iron accumulation in particular areas of the brain is an upstream cause or a downstream consequence, possibly related to age-related changes in brain metals homeostasis. Many mechanisms involved in AD development make it difficult to create treatments for the disease. This review focusing that APP 5′-UTR sequences indeed are a significant regulator of Aβ precursor expression in neural cells. AD is characterized by dysregulation of the brain’s iron levels and the proteins that bind them.

However, the mechanism of APP progression and Aβ buildup of Alzheimer’s pathogenesis can be better understood by learning from this failure. To decrease the gap between fundamental mechanistic research and clinical practice, translational models and methods that more closely resemble the biology of AD are also needed. Most of the current AD treatment regimen consists of symptomatic measures and is not curative. In addition to the more widely used acetylcholinesterase inhibitors (AChERs) and NMDA receptor antagonists, the market for AD treatments has seen significant advancements in the last five years, particularly with the introduction of additional Aβ targeting monoclonal antibodies.

Most clinical trials of therapies based on the conventional wisdom about the pathophysiology of AD have not produced very positive results. A new hypothesis that will be very important to further research may be established as a result of the growing understanding of the function that iron dysregulation play in the pathophysiology of AD. The ramifications of our finding of completely functional IRE stem-loops of the APP mRNA, which encode important proteins and the agents responsible for neurodegenerative disorders, are a crucial part of this review. APP synthesis can be modulated by targeting the functional IRE mRNA stem-loop encoded by the APP 5′-UTR. A unique strategy for the possible therapy of AD is the targeted regulation of APP gene expression by the adjustment of 5′-UTR sequence function, as doing so can improve protective brain iron balance and give anti-amyloid efficacy by changing APP translation. Novel treatment approaches may include iron-chelating compounds that selectively target the IRE in the 5′-UTR of the APP mRNA, thereby avoiding iron-induced toxicity, APP overproduction, and Aβ buildup.

Future studies aim to raise awareness of iron overload and aid in the development of mRNA-based medications to treat AD by elucidating the upstream and downstream roles of iron in AD pathogenesis, better understanding iron dysregulation, and uncovering the role of APP mRNA as a post-transcriptional regulator of amyloid protein synthesis. RNA sequence-directed translation inhibitors that enhance iron homeostasis and decrease amyloid formation in the brain may one day be evaluated clinically for AD treatment. Together, iron chelators and mRNA-based inhibitors may be able to reduce Aβ and stop AD-related neuron death.

## Data Availability

No new data was created. All data analyzed or generated during this study from previously published study. The author can provide the datasets used and/or analyzed in this article upon reasonable request.
